# Pseudoexfoliation: Normative Data and Associations. The Central India Eye and Medical Study

**DOI:** 10.1371/journal.pone.0076770

**Published:** 2013-10-21

**Authors:** Jost B. Jonas, Vinay Nangia, Arshia Matin, Krishna Bhojwani, Ajit Sinha, Anshu Khare, Shubhra Agarwal, Karishma Bhate

**Affiliations:** 1 Suraj Eye Institute, Nagpur, India; 2 Department of Ophthalmology, Medical Faculty Mannheim of the Ruprecht-Karls-University Heidelberg, Mannheim, Germany; Zhongshan Ophthalmic Center, China

## Abstract

**Purpose:**

To assess the prevalence of pseudoexfoliation (PEX) and its associations in a population-based setting.

**Design:**

Population-based, cross-sectional study.

**Methods:**

The Central India Eye and Medical Study included 4711 individuals. All study participants underwent a detailed ophthalmological examination. After medical pupil dilation, PEX was assessed by an experienced ophthalmologist using slit-lamp based biomicroscopy.

**Results:**

Slit lamp examination results were available for 4646 (98.6%) study participants with a mean age of 49.3±13.3 years (range: 30–100 years). PEX was detected in 87 eyes (prevalence: 0.95±0.10% (95%CI: 0.75, 1.15) of 69 subjects (prevalence: 1.49±0.18% (95%CI: 1.14, 1.83). PEX prevalence increased significantly (*P*<0.001) from 0% in the age group of 30–39 years, to 2.85±0.56% in the age group of 60–69 years, to 6.60±1.21% in the age group of 70–79 years, and to 12.3±4.11% in the age group of 80+ years. In multivariate analysis, PEX prevalence was associated with higher age (*P*<0.001; regression coefficient B:0.11; odds ratio (OR): 1.11 (95%CI: 1.09, 1.13)), lower body mass index (P = 0.001; B: −0.12; OR: 0.88 (95CI: 0.82, 0.95)) and higher diastolic blood pressure (P = 0.002; B: 0.02; OR: 1.03 (95%CI: 1.01, 1.04)). In the multivariate analysis, PEX was not associated with retinal nerve fiber layer cross section area (*P* = 0.76) and presence of open-angle glaucoma (*P* = 0.15).

**Conclusions:**

In a rural Central Indian population aged 30+ years, PEX prevalence (mean: 1.49±0.18%) was significantly associated with older age, lower body mass index and higher diastolic blood pressure. It was not significantly associated with optic nerve head measurements, refractive error, any ocular biometric parameter, nuclear cataract, early age-related macular degeneration and retinal vein occlusion, diabetes mellitus, smoking, and dyslipidemia.

## Introduction

Pseudoexfoliation (PEX) is a disorder which is characterized by the appearance of a fibrillary whitish material on the lens surface, the lens zonules, ciliary body and other parts of the anterior chamber [1], [2]. Linked to the lysyl oxidase-like-one (LOXL1) gene, PEX has been shown to be associated with changes in the extracellular matrix, involving the skin, extraocular muscles, heart, lung, liver, kidney, and meninges in addition to the eye [1]–[5]. It is clinically important since it is associated with an instability of the lens zonules during cataract surgery and late postoperative dislocation of the lens capsule including the intraocular lens, to mention only a few reasons [6], [7]. The reported prevalence of PEX varies from 0.2% to 30% in different study populations [8]–[27]. Previously, three studies reported the prevalence of PEX in South Indian populations [10], [13], [22]. Since the South Indian population with a mostly Dravidian background is ethnically different from the Central Indian population with a mostly Indo-Aryan background and since most previous studies were performed in urban regions or in rural regions with a relatively developed infrastructure, we conducted this study to assess the prevalence of PEX and to search for associations between PEX and other ocular and systemic parameters in the rural Central Indian population. Rural central India is one of the least developed regions in India with the lifestyle partially unchanged for the last 100 years.

## Methods

### Ethics Statement

The Medical Ethics Committee of the Medical Faculty Mannheim of the Ruprecht-Karls-University Heidelberg and a similar committee of the Suraj Eye Institute/Nagpur approved the study; all participants gave informed written consent, according to the Declaration of Helsinki.

The Central India Eye and Medical Study (CIEMS) is a population-based cross-sectional study in Central India. As described in detail recently, the study was performed in 8 villages in Kalmeshwar Tehsil, a rural region of Eastern Maharashtra at a distance of about 40 km from Nagpur in the geographical center of India [28]. The villages were chosen as locations for the study because they were located in a typical rural region of Central India, and were a relatively long distance from the nearest city (Nagpur). Of a total population of 13,606 villagers, 5885 subjects met the inclusion criterion of an age of 30+ years. There was no exclusion criterion. Of the 5885 eligible subjects, 4711 subjects (2191 men (46.5%)) participated, resulting in a response rate of 80.1%. The mean age was 49.5±13.4 years (median: 47 years; range: 30–100 years), and the mean reported monthly income was 1584±1233 rupees (1 US dollar equals roughly 50 rupees); the rate of illiteracy was 35%. Among the 1174 non-participants were 685 (58.3%) men; the mean age was 48.6±14.1 years (median: 45 years; range: 30–95 years). The group of study participants and the group of non-participants did not differ significantly in age (P = 0.06), while the proportion of men was significantly (*P*<0.001) higher in the group of non-participants.

All examinations were carried out at the hospital. Trained social workers filled out a questionnaire for the participants; this questionnaire included questions regarding socioeconomic background and living conditions, tobacco use and alcohol consumption, and any known diagnosis of major systemic diseases. Pulse, arterial blood pressure, and body height and weight were recorded. One-and-a-half hours after a standardized lunch, blood and urine samples were obtained and biochemically analyzed. Uncorrected visual acuity and visual acuity with the subjectś glasses and after refractive correction were measured using modified Early Treatment of Diabetic Retinopathy Study (ETDRS) charts (Light House Low Vision Products, New York, NY). Automated refractometry and subjective refraction were performed on all subjects independent of visual acuity. Visual field examinations were performed with frequency-doubling perimetry using the screening program C-20-1 (Zeiss-Humphrey, Dublin, CA). Intraocular pressure was measured by a slit lamp mounted Goldmann applanation tonometer; if the measurements were higher than 21 mmHg, tonometry was repeated. Slit lamp biomicroscopy was carried out by a fellowship-trained ophthalmologist, and any abnormality of the anterior segment was noted. Corneal pachymetry was performed by sonography using the Pacscan (Sonomed, Lake Success, NY). Ocular biometry was performed by partial coherence interferometry (IOL Master; Zeiss Co, Oberkochen, Germany). Anterior chamber depth, lens thickness and axial length were measured for both eyes of all subjects. Using the slit lamp, photographs of the limbal region were taken to assess the limbal anterior chamber depth at the most peripheral part of the cornea, as described by van Herick [29]. With the slit lamp beam set at an angle of 60° to the sagittal axis, the chamber depth was expressed as a percentage of the corneal thickness at that location. Additionally, the concept of Foster and colleagues was applied to assess the peripheral chamber depth in 6 grades ranging from 1 for “1–5% of limbal chamber depth”, 2 for “6–10% of limbal chamber depth, 3 for “11 to 25% of limbal chamber depth”, 4 for “26 to 50% of limbal chamber depth”, 5 for “51 to 75% of limbal chamber depth”, and 6 for “76 to 100% of limbal chamber depth” [30].

Gonioscopy was performed for all study participants in dim illumination using the magnaview single mirror gonio lens (Ocular Instruments, Bellevue, WA. USA). The slit beam was brought to its narrowest, and least height on a Haag Streit type slit lamp, to reduce the effect of light on the anatomy of the anterior chamber angle. The chamber angle was estimated as open, if in primary position the posterior pigmented part of the trabecular meshwork was visible without indentation [31], [32], [33]. There was appositional closure of the angle if, independently of the direction of gaze, the posterior trabecular meshwork could be seen only upon indentation. Synechial closure of the anterior chamber angle was determined on indentation gonioscopy. In subjects with any extent of occludable angles, indentation gonioscopy was performed with the Sussman 4 mirror goniolens (Ocular Instruments, Bellevue, WA. USA). The pupil was dilated using tropicamide 0.8% and phenylephrine 5% three times at 15 minute intervals so that all subjects attained maximal pupillary dilatation. A second slit lamp examination was performed to assess the presence of PEX. PEX was graded into stage 1 defined as faint PEX with small dark islands on the lens surface in the mid-peripheral annular region; stage 2 defined as confluencing islands in the mid-peripheral annular region; stage 3 defined as edge of PEX material clearly detectable on the lens surface at the peripheral border of the central island or at the central border of the peripheral region; stage 4 defined as circular edge of PEX material visible on the lens surface; stage 5 defined as PEX dandruff on the pupil margin; and stage 6 defined as massive PEX including PEX material on the retrocorneal surface. Digital photographs of the lens were taken and nuclear sclerosis was graded according to the Age Related Eye Disease Study [34]. Retro-illuminated photographs of the lens for assessment of cortical opacities were obtained using the Zeiss FF450 telecentric fundus camera (Zeiss Meditec Co., Oberkochen, Germany).

Digital monoscopic photographs of the optic disc (20 degrees; fundus camera type CR6-45NM, Canon Inc. U.S.A.) and macula (50 degrees) were taken. Magnification by optic media was corrected for by a built-in algorithm and we measured the area and horizontal diameter and vertical diameter of the optic disc and cup. Glaucoma was defined in two ways. First it was defined according to the criteria of the International Society of Geographical and Epidemiological Ophthalmology (ISGEO) classification scheme [26]. In that definition, criteria for a category 1 diagnosis (structural and functional evidence) were a vertical cup/disc diameter ratio (VCDR) or an inter-eye asymmetry in the VCDR of ≥97.5th percentile for the normal population, or a neuroretinal rim width reduced to ≤0.1 VCDR (between 11 to 1 o'clock or 5 to 7 o'clock), in addition to a definite visual field defect consistent with glaucoma. Criteria for the category 2 diagnosis (advanced structural damage with unproven visual field loss) were a VCDR or a VCDR asymmetry ≥99.5th percentile for the normal population. Criteria for a category 3 diagnosis (for eyes the optic nerve head of which could not be examined or for which a visual field examination was not possible) were a visual acuity <3/60 combined with either an intraocular pressure >99.5th percentile, or definite glaucoma medical records such as filtering surgery history. In a second step, glaucoma was diagnosed based on a glaucomatous appearance of the optic disc. The optic nerve head was glaucomatous (1) if the inferior-superior-nasal-temporal (ISNT)-rule of the neuroretinal rim shape was not fulfilled in early glaucoma and in eyes with a normally shaped optic disc (it included a notch in the neuroretinal rim in the temporal inferior region and/or the temporal superior region); or (2) if an abnormally large cup was present in a small optic disc which normally would not show cupping. The assessment of the optic disc photographs was carried in a masked manner without knowledge of intraocular pressure or the perimetric results. Each photograph of a glaucomatous optic disc was independently adjudicated by two senior graders (VN and JBJ). In the case that the two graders did not agree upon the diagnosis (what was the case in about 10% of the cases), the photographs were re-assessed in a third session by both graders together. In all of these cases, a mutual agreement was reached. In addition to the optic disc photographs, confocal laser scanning tomograms (HRT, Heidelberg Engineering, Heidelberg, Germany) of the optic disc were taken for all eyes. The whole glaucoma group was differentiated into subjects with open-angle glaucoma and with primary angle closure glaucoma. Open-angle glaucoma was characterized by an open anterior chamber angle, in addition to a normal depth of the anterior chamber as assessed by slit lamp biomicroscopy. In angle-closure glaucoma, the anterior chamber angle was occluded or occludable. The anterior chamber angle was defined as occludable, if >270° of the posterior trabecular meshwork (the part which is often pigmented) could not be seen upon static gonioscopy [31]–[33]. In addition, other features for angle-closure glaucoma were iris whirling and glaukomflecken in the anterior subcapsular lens region, in combination with a narrow anterior chamber angle. Mean ocular perfusion pressure was defined as: 2/3×(diastolic blood pressure +1/3×(systolic blood pressure – diastolic blood pressure)) – intraocular pressure.

Only those subjects with assessment of the presence of pseudoexfoliation were included into the study. Statistical analysis was performed using a commercially available statistical software package (SPSS for Windows, version 20.0, IBM-SPSS, Chicago, IL). In a first step, we determined the prevalence of pseudoexfoliation (presented as mean ± standard error). In a second step, we performed univariate analyses of the associations between the presence of pseudoexfoliation and other ocular and systemic parameters. In a the third step, we carried out binary regression analyses with the presence of pseudoexfoliation as the dependent parameter and all parameters as independent variables which were associated significantly with the presence of pseudoexfoliation in the univariate analyses. We then removed step by step all independent parameters for which the *P*-values were higher than 0.05, starting with the parameters with the highest *P*-values, until all remaining independent parameters were significantly associated with the presence of PEX. All *P*-values were two-sided. Odds ratios (OR) and 95% confidence intervals (CI) were presented.

## Results

Out of the 4711 subjects included in the study, results of the slit lamp examination for PEX were available for 9175 (97.4%) eyes of 4646 (98.6%) persons. The other 65 participants were excluded for assessment for PEX due to refusal for pupillary dilation or due to corneal opacities which rendered a slit examination of the lens surface impossible. The mean age of the 4646 participants (2484 (53.5%) women) was 49.3±13.3 years (median: 46 years; range: 30 to 100 years), and mean refractive error was −0.15±1.78 diopters (median: +0.00 diopters; range: −21.75 to +6.25 diopters), and mean axial length was 22.66±0.89 mm (median: 22.62 mm; range: 18.71 to 34.20 mm). The group of subjects with PEX assessment as compared with the group of subjects excluded for the PEX evaluation were significantly younger (*P*<0.001), more myopic (*P*<0.001), and had longer axial length (*P* = 0.02). Both groups did not differ significantly in gender (*P* = 0.78) and intraocular pressure (*P* = 0.06).

**Figure 1:**
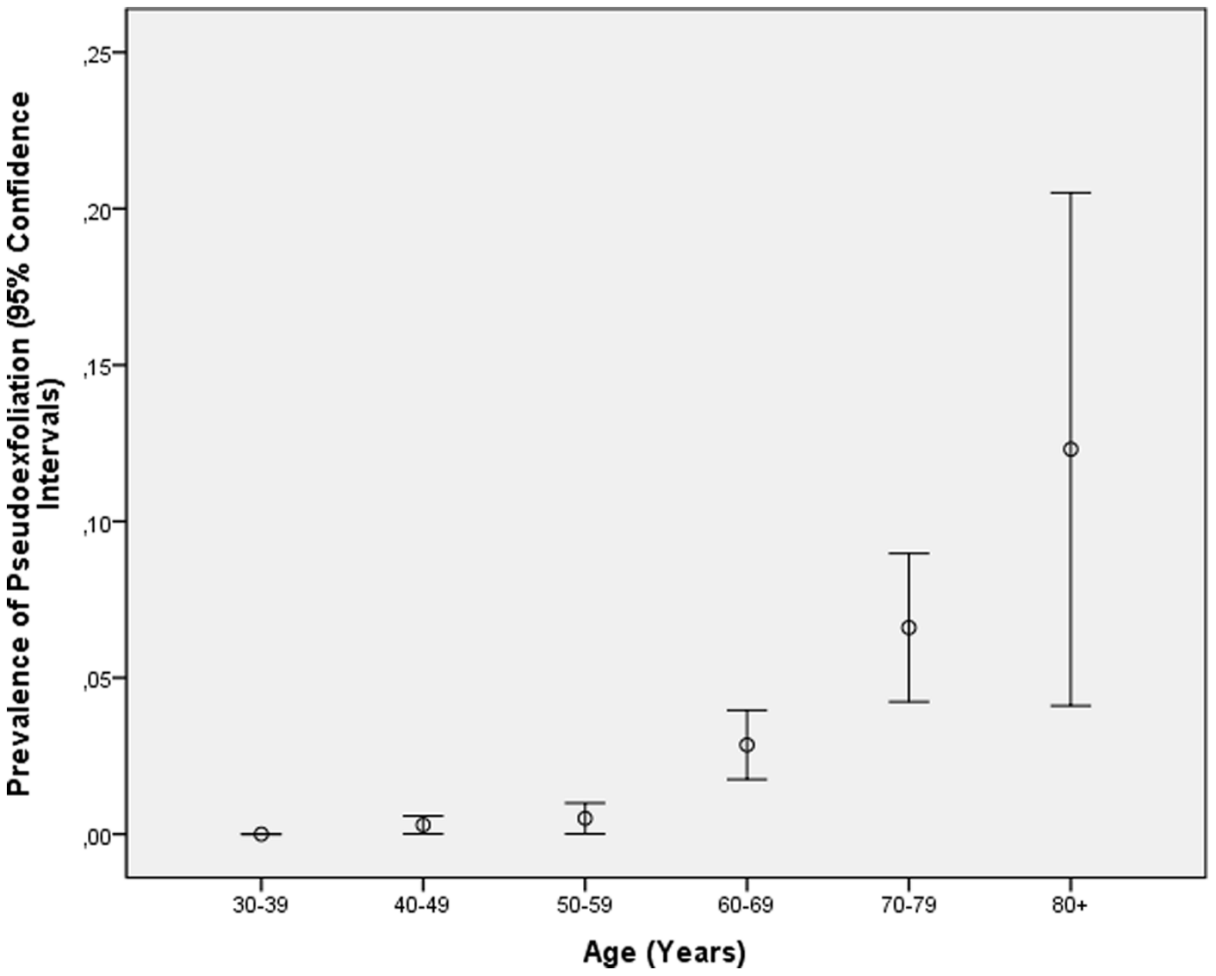
Diagram showing the prevalence of pseudoexfoliation in the Central India Eye and Medical Study, stratified by age.

PEX was detected in 87 eyes (prevalence: 0.95±0.10% (95%CI: 0.75, 1.15) in 69 subjects (prevalence: 1.49±0.18% (95%CI: 1.14, 1.83). PEX was found bilateral in 18/69 (26%) participants. There was no significant difference in the frequency of PEX in the right eye versus the left eye in the subjects with unilaterally detected PEX. The prevalence of PEX increased significantly (*P*<0.001) with age (Fig. 1), from 0% in the age group of 30–39 years, to 0.29±0.15% in the age group of 40–49 years, to 0.50±0.25% in the age group of 50–59 years, to 2.85±0.56% in the age group of 60–69 years, to 6.60±1.21% in the age group of 70–79 years, and to 12.3±4.11% in the age group of 80+ years. In a parallel manner, the mean PEX stage increased significantly (*P*<0.001) with older age. Taking only the population with an age of 40+ years, 50+ years, and 60+ years, the mean prevalence of PEX per eye and per subject was 1.25±0.13% (95%CI: 0.99, 1.51) and 1.96±0.23% (95%CI: 1.50, 2.41), 1.97±0.21% (95%CI: 1.55, 2.39) and 3.01±0.37% (95%CI: 2.29, 3.73), and 3.00±0.33% (95%CI: 2.35, 3.65) and 4.47±0.56% (95%CI: 3.37, 5.57), respectively.

In univariate analysis, the overall prevalence of pseudoexfoliation was significantly associated with the systemic parameter of higher age (*P*<0.001; OR: 1.11; correlation coefficient r = 0.17) (Fig. 1), lower body mass index (*P*<0.001; r = −0.06), lower height (*P* = 0.004; r = −0.04), lower body weight (*P*<0.001; r = −0.007), higher diastolic blood pressure (*P* = 0.03; r = −0.05), higher systolic blood pressure (*P*<0.001; r = 0.10), higher ocular perfusion pressure (*P*<0.001; r = 0.05), lower level of education (*P*<0.001; r = −0.08), and lower blood hemoglobin concentration (*P* = 0.02; r = −0.04); and with the ocular parameters of more myopic refractive error (*P* = 0.009; r = −0.03), higher degree of nuclear cataract (*P*<0.001; r = 0.11), higher intraocular pressure (*P* = 0.03; r = 0.02), lower mean retinal nerve fiber layer thickness (*P*<0.001; r = −0.04), and presence of open-angle glaucoma (*P*<0.001; OR: 7.60).

Presence of PEX was not significantly associated with the systemic parameters of gender (*P* = 0.23), smoking (*P* = 0.29), smoking package years (*P* = 0.73), blood concentration of high-density lipoproteins (*P* = 0.74), creatinine (*P* = 0.37), cholesterol (*P* = 0.20), postprandial blood concentration of glucose (*P* = 0.67), glycosylated hemoglobin HbA1c (*P* = 0.10), and presence of diabetes mellitus (*P* = 0.27); and with the ocular parameters of axial length (*P* = 0.56), central corneal thickness (*P* = 0.22), anterior corneal curvature radius (*P* = 0.36), anterior chamber depth (*P* = 0.12), lens thickness (*P* = 0.20), presence of angle-closure glaucoma (*P* = 0.72), optic disc area (*P* = 0.62), vertical cup/disc ratio (*P* = 0.20), horizontal cup/disc ratio (*P* = 0.05), neuroretinal rim area (*P* = 0.31), neuroretinal rim volume (*P* = 0.27), presence of diabetic retinopathy (*P* = 0.75), presence of early age-related macular degeneration (*P* = 0.84), presence of retinitis pigmentosa (*P* = 0.81), and presence of retinal vein occlusion (*P* = 0.61). Side differences in the presence of PEX were not significantly associated in side differences in intraocular pressure (*P* = 0.40).

In a multivariate analysis, which included presence of PEX as dependent variable and all those parameters as independent variables, which were significantly associated with PEX in the univariate analysis, presence of PEX remained to be significantly associated only with higher age (*P*<0.001; regression coefficient B: 0.11; OR: 1.11 (95%CI: 1.09, 1.13)), lower body mass index (*P* = 0.001; B: −0.12; OR: 0.88 (95%CI: 0.82, 0.95)), and higher diastolic blood pressure (*P* = 0.002; B: 0.02; OR: 1.03 (95%CI: 1.01, 1.04)), while it was no longer significantly associated with systolic blood pressure (*P* = 0.97), body height (*P* = 0.81), intraocular pressure (*P* = 0.80), retinal nerve fiber layer cross section area (*P* = 0.76), blood concentration of hemoglobin (*P* = 0.52), level of education (*P* = 0.98), degree of nuclear cataract (*P* = 0.39), refractive error (*P* = 0.32), and presence of open-angle glaucoma (as defined by ISGEO criteria) (*P* = 0.38) or presence of open-angle glaucoma (as defined by ophthalmoscopy) (*P* = 0.15). If visual acuity was added to the analysis, after adjusting for age, diastolic blood pressure and body mass index, PEX was associated with lower best corrected visual acuity (logMAR) (*P* = 0.004; B: 0.44; OR: 1.56 (95%: 1.15, 2.11).

In eyes with glaucoma (as defined by ISGEO criteria) (n = 129 eyes), PEX was more common, however not significantly more common (*P* = 0.20; OR: 2.54 (95%CI: 0.61, 10.5) than in the remaining eyes without glaucoma (1.6±1.1% (95%CI: 0.0, 3.7) versus 0.6±0.1% (95%CI: 0.5, 0.8)). In eyes with glaucoma (as defined by ophthalmoscopy) (n = 176 eyes), PEX was significantly more common than in the remaining eyes without glaucoma 4.0±1.5% (95%CI: 1.1, 6.9) versus 0.6±0.1% (95%CI: 0.5, 0.8); P<0.001; OR: 6.73 (95%CI: 3.01, 15.0). Differentiating within the glaucoma group between open-angle glaucoma and angle-closure glaucoma revealed significant differences in the prevalence of PEX for eyes with open-angle glaucoma versus the remaining eyes (*P*<0.001; OR: 7.60 (95%CI: 3.40, 17.0), while the difference in the PEX prevalence between eyes with angle closure glaucoma versus the remaining eyes was statistically not significant (*P* = 1.00). After adjustment for age, the correlation between open-angle glaucoma and PEX was no longer statistically significant *P* = 0.10).

If PEX was defined only as the severe stages 4 to 6 (characterized by circular edge of PEX material visible on the lens surface, PEX dandruff on the pupil margin, and massive PEX including PEX material on the retrocorneal surface), the presence of severe PEX was significantly associated with older age (*P*<0.001; B: 0.11; OR: 1.12 (95%CI: 1.09, 1.15)), lower body mass index (*P* = 0.005; B: −0.14; OR: 0.87 (95%CI: 0.80, 0.96)) and higher diastolic blood pressure (*P* = 0.005; B: 0.03; OR: 1.03 (95%CI: 1.01, 1.05)), but not with open-angle glaucoma (*P* = 0.20).

## Discussion

In our population-based study from rural Central India, the prevalence of PEX in 30+ subjects was 0.95±0.10% per eye 1.49±0.18% per subject. In the study population with an age of 40+ years, the mean prevalence of PEX per eye and per subject was 1.25±0.13% (95%CI: 0.99, 1.51) and 1.96±0.23% (95%CI: 1.50, 2.41), respectively. In multivariate analysis, PEX prevalence was associated with higher age, lower body mass index and higher diastolic blood pressure, while it was not significantly associated with intraocular pressure, retinal nerve fiber layer cross section area, any optic nerve head measurements and presence of open-angle glaucoma or angle-closure glaucoma, refractive error and any ocular biometric parameter, nuclear cataract, early age-related macular degeneration and retinal vein occlusion, diabetes mellitus, smoking, and dyslipidemia. Side differences in the presence of PEX were not significantly associated in side differences in intraocular pressure.

The PEX prevalence found in our study population was lower than the PEX prevalence reported from studies from South India (Aravind Comprehensive Eye Survey: age: 40+ years; PEX prevalence: 6.0% (95%CI: 5.3, 6.6) [10]; Tamil Nadu Chennai Glaucoma Study: age: 40+ years; 3.8% [22]; and Andhra Pradesh Eye Disease Study: age: 40+ years; 3.01%) [13], from Myanmar (age: 40+ years; PEX prevalence: 3.4%) [18], from Australia (PEX prevalence, in white Australians: 3.0%; indigenous Australians: age: 40+ years: 5.9%) [23], and from North China (age: 50+ years; PEX prevalence: 5.8%) [35]. The PEX prevalence in our study population was similar to the one from the Japanese Hisayama Study (50+ old population: PEX prevalence: 3.4%) [14]. The PEX prevalence in our study population was higher than in two previous studies on Chinese: a hospital based study from Hong Kong with a prevalence of 0.4% (age: 60–91 years) and the population-based Singaporean Tanjong Pagar Study with a prevalence of 0.2% [12], [36]. In studies from Scandinavian countries and from Greece, the prevalence of PEX was generally higher than in all other regions: In the population-based Reykjavik Eye Study, PEX prevalence was 10.7% with an increase from 2.5% in subjects aged 50–59 years to 40.6% in subjects aged 80+ years [16]. In a similar manner, PEX prevalence was 8.1% in a Finnish population [15]. In a Northern Swedish population aged 66 years, PEX prevalence was 23%. [17]. In the Greek Thessaloniki Eye Study on subjects aged 60+ years, PEX prevalence was 11.9% [21]. The differences in the reported prevalence of PEX may be due to differences in the study populations, in the detection technique and in the definition of PEX. To cite an example, the South Indian Aravind examined a Tamil Nadu population which by its Dravidian origin was ethnically different from our Indo-Arian study population in Central India. The Aravind Survey defined PEX by the presence of typical white deposits on the anterior lens surface with additional sites including the cornea, iris, anterior vitreous face, posterior capsule, and intraocular lens in cataract-operated eyes; the anterior chamber angle as assessed upon gonioscopy showed an increased pigmentation, PEX deposition, and PEX material within the angle structures. Interestingly, 25.7% of the subjects in the Aravind Study with PEX were bilaterally blind, with about 90% of this bilateral blindness caused by cataract. A recent study highlighted the dependence of PEX prevalence on geographic features. Kang and colleagues examined prospectively the association between demographic and geographic factors in relation to exfoliation glaucoma or exfoliation glaucoma suspect as part of the Health Professionals Follow-up Study [24]. They found that exfoliation glaucoma or exfoliation glaucoma suspect was strongly related with age. They did not find any predisposition to exfoliation glaucoma by ancestry, particularly Scandinavian ancestry. Compared with a lifetime of living in the northern tier of the continental United States, lifetime residence in the middle geographic tier and in the southern geographic tier was associated with markedly reduced risks of exfoliation glaucoma or exfoliation glaucoma suspect. They concluded that in their mainly white study population from the United States, living in the middle or southern regions of the United States relative to living in the northern region was associated with a reduced risk of exfoliation glaucoma. In a retrospective study of 626,901 eye care recipients, northern-tier residence (above 42°N latitude) was associated with an increased hazard of PEX (adjusted hazard ratio [HR], 2.14), and Southern-tier (below 37°N latitude) was associated with a reduced hazard of PEX (HR, 0.83) [37]. After adjustment for joint environmental effects, for every 1° increase in July high temperature, the hazard of PEX decreased by 9% (HR: 0.91); for every 1° increase in January low temperature, the hazard decreased 3% (HR: 0.97). For each additional sunny day annually, the hazard increased by 1.5% (HR: 1.02) in locations with average levels of other climatic factors. The authors concluded that ambient temperature and sun exposure may be important environmental triggers of PEX.

The prevalence of PEX increased significantly with age (Fig. 1), in accordance with all previous reports [6], [8]–[27]. In our study population, PEX prevalence increased from 0% in the age group of 30–39 years, to 0.29±0.15% in the age group of 40–49 years, to 0.50±0.25% in the age group of 50–59 years, to 2.85±0.56% in the age group of 60–69 years, to 6.60±1.21% in the age group of 70–79 years, and to 12.3±4.11% in the age group of 80+ years. In our study, PEX prevalence was not significantly associated with gender. It was in accordance with the South Indian Chennai glaucoma study, the Japanese Hisayama study, an Australian study by McCarty and colleagues, and a recent evaluation in the Beijing Eye Study [9], [14], [22]. It is in disagreement with the Framingham Eye Study, the Reykjavik Eye Study, and the study by Astrom and coworkers in which PEX was significantly more prevalent in women than in men [16], [17], [38]. In the Aravind Comprehensive Eye Survey, PEX prevalence was higher in men than in women [10].

In univariate analysis, eyes with ophthalmoscopic glaucoma as compared to non-glaucomatous eyes showed significantly more often PEX (4.0±1.5% (95%CI: 1.1, 6.9) versus 0.6±0.1% (95%CI: 0.5, 0.8); *P*<0.001; OR: 6.73 (95%CI: 3.01, 15.0). This was valid for open-angle glaucoma (*P*<0.001) but not for angle-closure glaucoma (*P* = 1.00). In multivariate analysis with adjustment for age, the association between PEX and glaucomatous optic nerve damage was no longer statistically significant. Correspondingly, in multivariate analysis, prevalence of PEX was not significantly associated with parameters of glaucomatous optic neuropathy such as small neuroretinal rim area and high vertical cup/disc diameter ratios. These findings partially agree with, and partially contradict, the results of other studies. In the South Indian Andhra Pradesh Eye Disease Study, the prevalence of PEX in subjects with glaucoma was 4.2% (95% CI: 0.17–8.23) what was not markedly different from the prevalence of PEX in the general study population with a PEX prevalence of 3.01% in subjects aged 40+ years and 6.28% in subjects with an age of 60+ years [13]. In the Aravind Comprehensive Eye Survey, glaucoma was diagnosed in 23 (7.5%) of the 308 persons with PEX who had a mean age of about 61 years (63.7 years for men; 58.1 years for women) [10]. In univariate analysis, the odds ratio for having glaucoma was greater among the subjects with PEX than the subjects without PEX. This association however was not adjusted for age. In the Beijing Eye Study, PEX was not clearly associated with the prevalence of glaucoma [35]. In the South African study by Rotchford and colleagues, the age-adjusted and gender-adjusted odds ratios for the association between open-angle glaucoma and PEX had marginal statistical significance and were 2.3 (95% CI: 1.0–5.2) and 2.8 (95% CI: 1.2–6.3) for two study populations [11]. In the Thessaloniki Eye Study, the prevalence of glaucoma among subjects with PEX was 15.2% and the prevalence of glaucoma among subjects without PEX was 4.7% [26]. In multivariate analysis restricted to persons who participated in clinic visits, PEX (OR: 2.81) in addition to intraocular pressure (OR: 1.21 per 1 mm Hg), history of coronary artery bypass or vascular surgery (OR: 1.95) and moderate-to-high myopia (≥−3 diopters; OR: 2.40) were factors associated with higher odds for open-angle glaucoma [27]. In the Australian study by McCarty and Taylor, glaucoma was clearly related to PEX (OR: 3.80 (95% CI = 1.73, 8.33) after adjusting for age and cataract [9].

PEX was significantly (*P* = 0.004) associated with lower best corrected visual acuity in our study after adjusting for age, diastolic blood pressure and body mass index. It is in agreement with other studies from India such as the Andhra Pradesh Eye Disease Study and the Aravind Comprehensive Eye Survey, in which PEX was significantly associated with blindness after adjustment for age [10], [13]. In our study, PEX was significantly associated with higher diastolic blood pressure confirming the Japanese Hisayama study [14]. The meaning of this finding has remained unclear.

Potential limitations of our study should be mentioned. First, a major concern in any prevalence study on PEX is the method of its detection. In our study, the pupils were dilated using tropicamide 0.8% and phenylephrine 5% three times at 15 minute intervals so that almost all subjects attained maximal pupillary dilatation. The slit lamp examination was performed by an experienced ophthalmologist who was specially trained for, and focused on, the detection of PEX. Second, nonparticipation is a concern for any population-based study. The Central India Eye and Medical Study had a reasonable response rate of 80.1%, however, differences between participants and non-participants could have led to a selection artifact. Third, the LOXL1 gene variants, which was reported as the associated gene with PEX, was not tested in our research [5].

In conclusion, in a rural Central Indian population aged 30+ years, PEX prevalence (mean: 1.49±0.18%) was significantly associated with older age, lower body mass index and higher diastolic blood pressure, but not with intraocular pressure, optic nerve head measurements and glaucoma, refractive error and any ocular biometric parameter, nuclear cataract, early age-related macular degeneration and retinal vein occlusion, diabetes mellitus, smoking, and dyslipidemia,.
